# Palladium Supported on Titanium Carbide: A Highly Efficient, Durable, and Recyclable Bifunctional Catalyst for the Transformation of 4-Chlorophenol and 4-Nitrophenol

**DOI:** 10.3390/nano8030141

**Published:** 2018-03-02

**Authors:** Guangyin Fan, Xiaojing Li, Caili Xu, Weidong Jiang, Yun Zhang, Daojiang Gao, Jian Bi, Yi Wang

**Affiliations:** 1 College of Chemistry and Materials Science, Sichuan Normal University, Chengdu 610068, China; lixiaojing@sicnu.edu.cn (X.L.); cailixu@sicnu.edu.cn (C.X.); daojianggao@sicnu.edu.cn (D.G.); bijian686@sicnu.edu.cn (J.B.); yiwang@sicnu.edu.cn (Y.W.); 2 School of Chemical and Environmental Engineering, Sichuan University of Science & Engineering, Zigong 643000, China; jwdxb@suse.edu.cn

**Keywords:** palladium, titanium carbide, catalytic transformation, 4-chlorophenol, 4-nitrophenol

## Abstract

Developing highly efficient and recyclable catalysts for the transformation of toxic organic contaminates still remains a challenge. Herein, Titanium Carbide (Ti_3_C_2_) MXene modified by alkali treatment process was selected as a support (designated as alk-Ti_3_C_2_X_2_, where X represents the surface terminations) for the synthesis of Pd/alk-Ti_3_C_2_X_2_. Results show that the alkali treatment leads to the increase of surface area and surface oxygen-containing groups of Ti_3_C_2_X_2_, thereby facilitating the dispersion and stabilization of Pd species on the surface of alk-Ti_3_C_2_X_2_. The Pd/alk-Ti_3_C_2_X_2_ catalyst shows excellent catalytic activity for the hydrodechlorination of 4-chlorophenol and the hydrogenation of 4-nitrophenol in aqueous solution at 25 °C and hydrogen balloon pressure. High initial reaction rates of 216.6 and 126.3 min^−1^·$g_{\mathrm{pd}}^{-1}$ are observed for the hydrodechlorination of 4-chlorophenol and hydrogenation of 4-nitrophenol, respectively. Most importantly, Pd/alk-Ti_3_C_2_X_2_ exhibits excellent stability and recyclability in both reactions without any promoters. The superior property of Pd/alk-Ti_3_C_2_X_2_ makes it as a potential material for practical applications.

## 1. Introduction

Noble metal nanoparticles have been intensively investigated as catalysts for a variety of organic catalytic reactions. It is well known that the structure and surface properties of the supports significantly affect the catalytic activity, chemical and environmental stability, and recyclability of the catalysts. Owing to its abundant surface chemistry, two-dimensional (2D) graphene has captured considerable attention as a promising matrix for nanoparticle stabilization of metal in many organic catalytic reactions [1,2,3,4]. Although graphene-supported catalysts have shown superior properties, the preparation procedures of graphene are tedious and time-consuming, which impede production scale-up. For practical applications, it is highly desirable to synthesize efficient catalysts by using carriers that can be conveniently prepared on a large scale. Graphene-like 2D transition metal carbides, designated as MXenes, are such materials, with the advantages of excellent electrical conductivity and good structural and chemical stability [5]. As an essential member of the MXenes group, Ti_3_C_2_ with its tailorable physicochemical properties and high physical and chemical stability can be easily synthesized on the large scale by etching the Al layers of Ti_3_AlC_2_ in hydrofluoric acid (HF) solution at room temperature. Ti_3_C_2_ not only has a graphene-like 2D layered structure, but also possesses abundant surface terminations because of the insertion of oxygen-containing groups (–OH and –COOH) into the inter-lamellar spaces of Ti_3_C_2_ after the removal of the Al atoms. Owing to its unique structure and surface properties, Ti_3_C_2_ has recently attracted widespread research interest [6,7,8,9]. For example, Xie and co-workers found that the Pt/Ti_3_C_2_X_2_ (X represents the surface terminations) catalyst showed better electrocatalytic performance than a commercial Pt/C catalyst in an oxygen reduction reaction [6]. Zhang et al. also discovered that a MXene/Ag_0.9_Ti_0.1_ composite had excellent electrocatalytic activity for the oxygen reduction reaction [7]. Satheeshkumar et al. reported the synthesis of Ag, Au, and Pd@MXene hybrids and used them as substrates for surface-enhanced Raman spectroscopy (SERS) [8]. Zou et al. showed that Ag/MXene could also be used as an anode material for lithium-ion batteries with long cycle lifetime and large capacity [9]. In previous studies, we also have shown that the Ti_3_C_2_X_2_-supported metal nanoparticles have exhibited outstanding catalytic properties toward the hydrolysis of ammonia borane and hydrodechlorination (HDC) of 4-chlorophenol (4-CP) under mild conditions [10,11,12,13]. For example, Pt_y_Co_1-y_/Ti_3_C_2_X_2_ (X = O, F) and RuNi nanoparticles stabilized on Ti_3_C_2_X_2_ (X = OH and/or F) catalysts have been identified as efficient catalysts for the hydrolysis of ammonia borane [11,12]. Rh/alk-Ti_3_C_2_X_2_ (X = O, F) has also been demonstrated as an active catalyst for HDC of 4-CP in a base-free aqueous medium [13]. However, the catalytic activity of Rh/alk-Ti_3_C_2_X_2_ was not satisfactory and the catalyst was not cost-effective considering the use of high-cost Rh as the active sites. It is plausible to explore the applications of Ti_3_C_2_ MXene as a support for developing highly active and cost-saving metal catalysts.

4-CP and 4-nitrophenol (4-NP) are considered priority pollutants for the environment and human beings because of their high toxicity and poor biodegradability [14]. Among the approaches to the disposal of 4-CP and 4-NP, catalytic hydrogenation has been identified as one of the most promising approaches because of its high efficiency and environmental friendliness. Moreover, the resulting products including phenol and 4-aminophenol (4-AP) are useful chemicals that can be applied in various applications [15]. Considerable research efforts have been devoted to developing supported catalysts with Pd nanoparticles as the active sites for HDC and hydrogenation reactions [14,15,16,17,18,19,20,21,22,23,24,25,26,27]. However, one limitation of the HDC of 4-CP reaction is that the catalysts suffer from serious deactivation by HCl, which is generated as a byproduct during the HDC process through the scission of C–Cl bonds in 4-CP. Therefore, a large amount of base additives such as NaOH and trimethylamine are required to maintain their catalytic activities [28,29,30,31]. Unfortunately, the additives may induce secondary pollution and extra work is needed to separate these bases from the reaction mixture. Regarding the disposal of 4-NP, most current present processes require a strong reducing agent such as NaBH_4_. Design and facile synthesis of stable, recyclable, and highly active catalysts for the transformation of 4-CP and 4-NP under mild conditions without additive or chemical reducing agent is highly desirable, considering its significant industrial importance.

So far, little attention has been devoted to investigating the catalytic performance of Ti_3_C_2_X_2_-supported Pd-based catalysts for the transformation of hazardous organic contaminants. Herein, we aim at developing a bifunctional catalyst with high catalytic activity, durability, and recyclability for the transformation of 4-CP and 4-NP in an aqueous solution without any additives. Specifically, alk-Ti_3_C_2_X_2_ was selected as the support to synthesize Pd/alk-Ti_3_C_2_X_2_ catalyst using a facile approach. Catalytic performance of the as-prepared catalyst was investigated for the HDC of 4-CP and the hydrogenation of 4-NP at room temperature in water. The recyclability of Pd/alk-Ti_3_C_2_X_2_ in both reactions was also tested. The current catalytic systems possess several useful characteristics compared with the reported Pd-based catalysts. Firstly, alk-Ti_3_C_2_X_2_ was firstly used as a support to deposit Pd nanoparticles by using EG as the reducing agent. It is discovered that the presence of oxygen-containing groups not only facilitates the anchoring of Pd nanoparticles but also improves the hydrophilicity of the catalyst, which is beneficial to the dispersion of the catalyst in aqueous phase. Secondly, the Pd/alk-Ti_3_C_2_X_2_ catalyst has a very high catalytic activity toward HDC of 4-CP at a high 4-CP concentration without any additives. The strong deactivation resistance of Pd/alk-Ti_3_C_2_X_2_ for HDC of 4-CP reaction may attribute to the strong interaction between the metal and the support. In addition, this catalyst also showed a very high activity toward the hydrogenation of 4-NP with hydrogen balloon pressure without additional strong reducing agents such as NaBH_4_, which is a cost-effective and green process. Finally, the Pd/alk-Ti_3_C_2_X_2_ catalyst is quite stable and can be recycled at least seven times in the absence of additives. The high stability and recyclability of the Pd/alk-Ti_3_C_2_X_2_ catalyst, especially for HDC of 4-CP, have never been reported so far. The high catalytic performance and recycling efficiency of the Pd/alk-Ti_3_C_2_X_2_ catalyst make it as a promising bi-functional material in HDC and hydrogenation reactions.

## 2. Materials and Methods 

### 2.1. Materials

Sodium tetrachloropalladate (Na_2_PdCl_4_·6H_2_O) was purchased from the Kunming Institute of Precious Metals, Kunming, China. Ethylene glycol (EG, >99%), absolute ethanol, concentrated HF (>49%), and sodium hydroxide (NaOH) were bought from Aladdin Industrial Inc., Shanghai, China. Hydrogen gas with a purity of 99.999% was supplied by Chengdu Taiyu Gas Co. Ltd., Chengdu, China. All reagents were used directly as received and without further purification. Ultrapure deionized water (18.2 MΩ) was used in all experiments.

### 2.2. Characterization

Brunauer–Emmett–Teller (BET) surface areas were measured by nitrogen adsorption at 77 K on an ASAP-2020 adsorption apparatus. Scanning electron microscopy (SEM) was carried out using a JSM-6510 (Rigaku, Osaka, Japan) operating at an accelerating voltage of 20 kV. Morphology and microstructure of Pd/alk-Ti_3_C_2_X_2_ were characterized by transmission electron microscopy (TEM, Tecnai G20, FEI, Hillsboro, OR, USA) operating at an accelerating voltage of 200 kV. X-ray diffraction (XRD) characterization data were collected on a PANalytical X’eprt diffractometer (Egham, Surrey, UK) with Cu Kα radiation (40 kV, 40 mA) from 5° to 50°. X-ray photoemission spectroscopy (XPS) was performed on a Thermo ESCALAB 250 Axis Ultra spectrometer (Thermo, Waltham, MA, USA) using a monochromatic Al Kα (hν = 1486.6 eV). ICP-OES analysis was carried out on SPECTRO ARCOS spectrometer (SPECTRO, Kleve, Germany). The removal efficiency of the catalyst for 4-CP was analyzed by gas chromatography (GC, Agilent 7890A, Agilent Technologies, Santa Clara, CA, USA) with a flame ionization detector (FID) detector and PEG-20 M capillary column (30 m × 0.25 mm, 0.25 μm film) and nitrogen was used as a carrier gas.

### 2.3. Synthesis of Ti_3_C_2_X_2_, alk-Ti_3_C_2_X_2_, and Pd/alk-Ti_3_C_2_X_2_

Ti_3_C_2_X_2_ was synthesized through the etching of the Al layers of Ti_3_AlC_2_ in a concentrated HF solution at 25 °C. Typically, Ti_3_AlC_2_ (4.0 g) was slowly added into a 250-mL beaker containing a concentrated HF solution (HF: 49%, 40 mL). Then, the mixture was continuously stirred at 25 °C for 24 h to remove the Al layers of Ti_3_AlC_2_. The suspension was treated by centrifugation and the resulting black solid was washed with water and absolute ethanol several times. Finally, the product was dried under a vacuum at 60 °C for 12 h. Alk-Ti_3_C_2_X_2_ was prepared according to the following procedures. The as-prepared Ti_3_C_2_X_2_ (0.3 g) was transferred into a 500-mL two-necked round-bottomed flask containing NaOH (15.0 g) and distilled water (300 mL). The mixture was stirred at 25 °C for 6 h and then separated by centrifugation. The resulting solid was treated by several cycles of washing with water and ethanol. Finally, the collected product was dried in a vacuum at 60 °C for 12 h.

The Pd/alk-Ti_3_C_2_X_2_ catalyst was synthesized as follows: alk-Ti_3_C_2_X_2_ (0.3 g), NaOH (0.1 g), and Na_2_PdCl_4_·6H_2_O (0.0438 g) were added into 20 mL of EG. After being treated by sonication for 1 h, the mixture was heated to 140 °C and maintained at this temperature for 4 h. The obtained precipitate was separated by centrifugation and washed several times with ethanol. Finally, the resultant catalyst was dried in a vacuum at 60 °C for 12 h. The Pd loading, determined by inductively coupled plasma (ICP), was around 4.4 wt %.

### 2.4. Activity Tests

The catalytic activity of Pd/alk-Ti_3_C_2_X_2_ was investigated for the HDC of 4-CP at 25 °C and hydrogen balloon pressure. In a typical reaction process, 5.0 mg of Pd/alk-Ti_3_C_2_X_2_ and 5 mL of aqueous solution of 4-CP (4-CP concentration: 2.5 g/L) were transferred into a 25-mL two-necked round-bottomed flask equipped with a hydrogen balloon. The flask was treated in a vacuum and then flushed with pure hydrogen several times. The reaction temperature was controlled at 25 °C by a water bath. During the reaction process, samples were withdrawn by a syringe at an interval of 10 min. The catalytic process for 4-NP hydrogenation was similar with 4-CP HDC except that 4-NP was used as the substrate. To test the recyclability, the Pd/alk-Ti_3_C_2_X_2_ catalyst was separated by centrifugation after each recycling run. The collected solid was washed with water and ethanol repeatedly and directly used for the next run. In all tests, the conversions for 4-CP and 4-NP catalyzed by Pd/alk-Ti_3_C_2_X_2_ were analyzed by GC (Agilent 7890A, Agilent Technologies, Santa Clara, CA, USA).

## 3. Results and Discussion

### 3.1. Characterization of the Catalyst

**Scheme 1:**
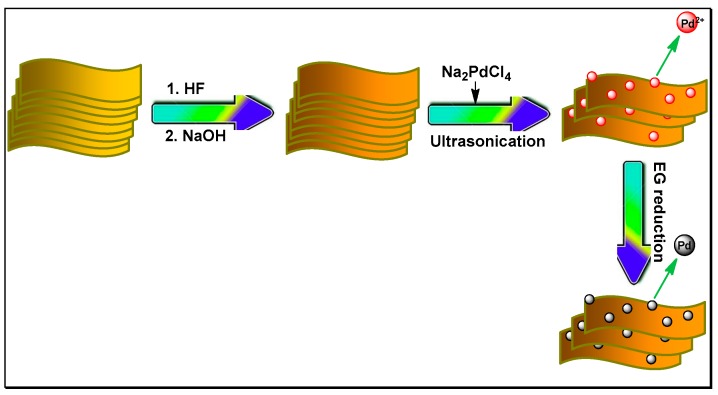
The procedure for the synthesis of Pd/alk-Ti_3_C_2_X_2_.

As shown in Scheme 1, the facile synthesis of Pd/alk-Ti_3_C_2_X_2_ was realized by using alk-Ti_3_C_2_X_2_ as a carrier and Na_2_PdCl_4_ as a precursor. First, the pristine Ti_3_AlC_2_ was soaked in concentrated HF at room temperature to produce Ti_3_C_2_X_2_. The resulting Ti_3_C_2_X_2_ was delaminated by sonication in aqueous solution to separate the thick-layered Ti_3_C_2_X_2_. Ti_3_C_2_X_2_ was further delaminated to alk-Ti_3_C_2_X_2_ through the alkalization treatment of Ti_3_C_2_X_2_ in NaOH aqueous solution. Subsequently, Pd ions were attached on the alk-Ti_3_C_2_X_2_ surface through coordination with oxygen-containing groups such as hydroxyl groups. Pd nanoparticles were anchored on the Ti_3_C_2_X_2_ surface through the reduction of Pd ions using ethylene glycol as the reducing agent.

**Figure 1:**
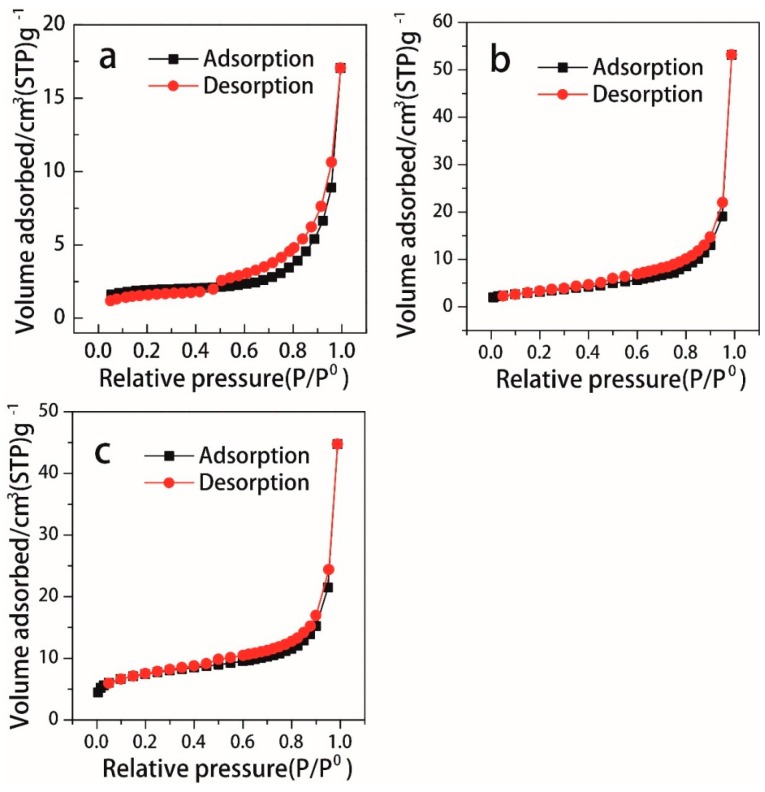
N_2_ adsorption–desorption isotherms of (**a**) Ti_3_C_2_X_2_, (**b**) alk-Ti_3_C_2_X_2_, and (**c**) Pd/alk-Ti_3_C_2_X_2_.

The specific surface areas of Ti_3_C_2_X_2_, alk-Ti_3_C_2_X_2_, and Pd/alk-Ti_3_C_2_X_2_ were determined by BET measurement and the results are shown in Figure 1. It is noteworthy that Ti_3_C_2_X_2_ has a surface area of 6.1 m^2^·g^−1^ because of its stacked structure (Figure 1a). Interestingly, the surface area of alk-Ti_3_C_2_X_2_ (11.5 m^2^·g^−1^) is about twice that of the pristine Ti_3_C_2_X_2_ after the alkalization process (Figure 1b), which is probably attributed to the increase of interlayer spacing because of the intercalation of Na ions [5]. The surface area of Pd/alk-Ti_3_C_2_X_2_ further increased to 24.6 m^2^·g^−1^ because of the attachment of Pd nanoparticles on the layered Ti_3_C_2_X_2_ surface (Figure 1c).

**Figure 2:**
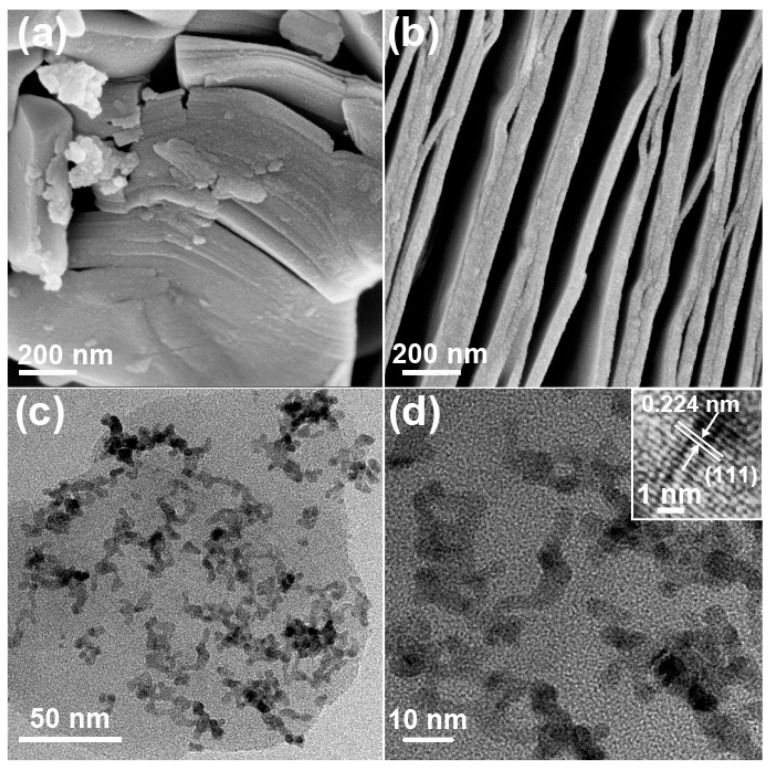
(**a**) SEM image of Ti_3_AlC_2_; (**b**) SEM image of Ti_3_C_2_X_2_; (**c**,**d**) TEM images of Pd/alk-Ti_3_C_2_X_2_ at different magnifications (the inset is the HRTEM image of Pd nanoparticles).

To identify the etching of Al layers in Ti_3_AlC_2_ using HF as a reducing agent, the morphology of the pristine Ti_3_AlC_2_ and Ti_3_C_2_X_2_ was first examined by SEM. As illustrated in Figure 2a, the pristine Ti_3_AlC_2_ displays a closely aligned layered structure. As shown in Figure 2b, the successful exfoliation of Ti_3_AlC_2_ was authenticated through observing the detriment of the closely aligned layered structure in a concentrated HF solution. The morphology of Pd on alk-Ti_3_C_2_X_2_ surface was characterized by the TEM technique and the results are shown in Figure 2c,d. Pd nanoparticles with a worm-like morphology are observed on the surface of alk-Ti_3_C_2_X_2_ when using Na_2_PdCl_4_ as a precursor and EG as a solvent and reducing agent. The high-resolution TEM (HRTEM) image shows a typical crystal plane with lattice spacing of 0.224 nm, which corresponds to the (111) plane of Pd [32].

**Figure 3:**
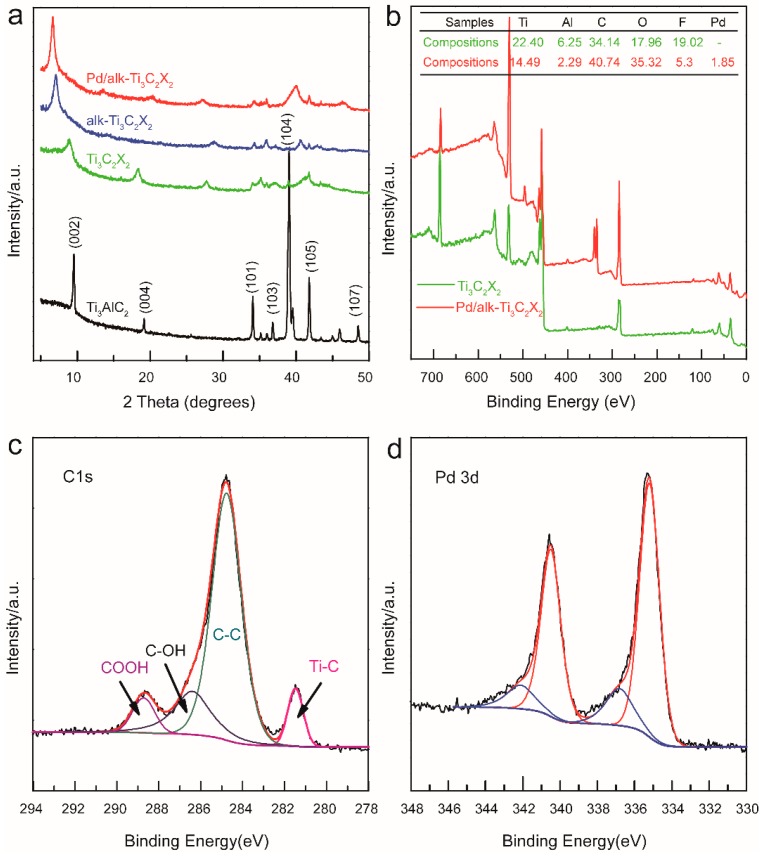
(**a**) XRD patterns of Ti_3_AlC_2_, Ti_3_C_2_X_2_, alk-Ti_3_C_2_X_2_, and Pd/alk-Ti_3_C_2_X_2_; (**b**) XPS survey scans of Ti_3_C_2_X_2_ and Pd/alk-Ti_3_C_2_X_2_ (Inset is the atomic ratio of the detected elements); (**c**,**d**) High-resolution XPS spectra of C1s and Pd 3d for Pd/alk-Ti_3_C_2_X_2_.

XRD patterns of Ti_3_AlC_2_, Ti_3_C_2_X_2_, alk-Ti_3_C_2_X_2_, and Pd/alk-Ti_3_C_2_X_2_ were recorded to investigate the structure of the selected materials. As shown in Figure 3a, the strong and sharp diffraction peaks match well with the standard card of Ti_3_AlC_2_ (JCPDS no. 52-0875), indicating the high purity of Ti_3_AlC_2_. The characteristic peaks of Ti_3_AlC_2_ disappear when Ti_3_AlC_2_ is treated with a concentrated HF aqueous solution. Consequently, several new peaks at 2θ = 9.0°, 18.5°, and 27.5° are observed because of the insertion of OH and F functional groups after the removal of Al atoms [6]. The observation of weak and broad peaks in Ti_3_C_2_X_2_ is attributed to the introduction of oxygen-containing functional groups on the surface of Ti_3_C_2_X_2_ after HF etching, indicating successful synthesis. It is noteworthy that the (002) diffraction peak of Ti_3_C_2_X_2_ shifts to a low angle after the alkalization process, owing to the occurrence of intercalation of Na ions [5]. In addition, no peaks corresponding to Pd or Pd oxide are detected by XRD characterization because of the low loading of Pd on the surface of alk-Ti_3_C_2_X_2_. It should be pointed out that the (002) peak position of Pd/alk-Ti_3_C_2_X_2_ further shifts toward a low angle, implying that Pd intercalation occurs. Compared with the pristine Ti_3_AlC_2_, the disappearance of the broad and strong diffraction peaks and the observation of new broad and weak peaks together with their slight shifts to a low angle verify the successful synthesis of Pd/alk-Ti_3_C_2_X_2_. Additionally, the XRD results also suggest that the crystal structure of alk-Ti_3_C_2_X_2_ does not change obviously during the reduction process.

The surface compositions of Ti_3_C_2_X_2_ and Pd/alk-Ti_3_C_2_X_2_ were measured by XPS surface survey scans. As shown in Figure 3b, signals corresponding to titanium, carbon, aluminum, oxygen, and fluorine are distinctively observed. A characteristic peak corresponding to Pd is detected in the Pd/alk-Ti_3_C_2_X_2_ sample, showing that Pd has been successfully anchored on the surface of alk-Ti_3_C_2_X_2_ with a surface atomic ratio of 1.85%. It should be pointed out that the detection of high fluorine content confirmed the insertion of F into the layers of Ti_3_C_2_ via the reaction of Ti_3_C_2_ with HF (Ti_3_C_2_ + 2HF = Ti_3_C_2_F_2_ + H_2_) when the Al layers were removed [33]. Significantly, the F groups could be replaced by OH groups and resulted in a decrease of F content after alkalization treatment. These results show that the alkalization of Ti_3_C_2_X_2_ benefited from the transformation of F functional groups to hydroxyl groups during the alkalization process [5,34]. The oxygen-containing groups favor the stabilization of Pd nanoparticles on the surface of alk-Ti_3_C_2_X_2_, thereby improving the hydrophilicity of Pd/alk-Ti_3_C_2_X_2_. The electronic states of C and Pd on alk-Ti_3_C_2_X_2_ surface were further analyzed by high-resolution XPS measurement. As shown in Figure 3c, the spectrum of C1s in Pd/alk-Ti_3_C_2_X_2_ sample can be fitted into four peaks with binding energies of 281.5, 284.8, 286.4, and 288.7 eV, which are attributed to various types of carbon atoms including Ti–C, C–C, C–OH, and HO–C=O, respectively [35]. These results verify the presence of oxygen-containing functional groups on the Ti_3_C_2_X_2_ surface, which is coincident with XRD results. The binding energy of Pd3d level was analyzed after the immobilization of Pd particles on the alk-Ti_3_C_2_X_2_ surface (Figure 3d). The two peaks with the binding energies of 335.2 and 340.3 eV are assigned to 3d_5/2_ and 3d_3/2_ of metallic Pd°. Notably, the Pd3d peak also presents two small peaks with binding energies of 337.1 and 342.3 eV, which correspond to the 3d5/2 and 3d3/2 of Pd^n+^ species.

### 3.2. Catalytic HDC of 4-CP

**Scheme 2:**
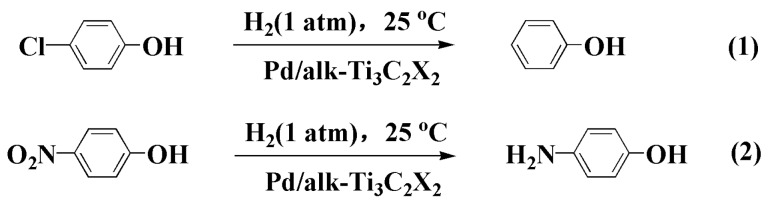
Reaction pathways for the HDC of 4-CP (1) and hydrogenation of 4-NP (2).

**Figure 4:**
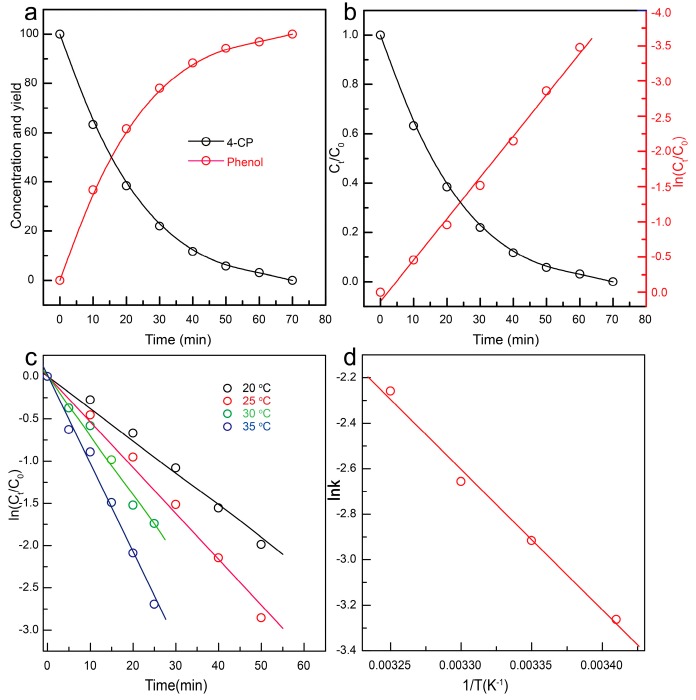
(**a**) Time course of evolution of the yield of phenol and conversion of 4-CP catalyzed by Pd/alk-Ti_3_C_2_X_2_ catalyst at 25 °C; (**b**) Plots of C_t_/C_0_ and ln (*C*_t_/*C*_0_) vs. time for the HDC of 4-CP; (**c**,**d**) Plots of ln (*C*_t_/*C*_0_) vs. time at different temperatures and the Arrhenius plots.

The catalytic performance of the as-synthesized Pd/alk-Ti_3_C_2_X_2_ catalyst was first studied for the HDC of 4-CP in a base-free medium at 25 °C and hydrogen balloon pressure. Regarding the reaction pathway shown in Scheme 2(1), hydrogen is absorbed and activated by the active sites of Pd on the surface of alk-Ti_3_C_2_X_2_. Secondly, the activated hydrogen provides two active hydrogen atoms by the cleavage of the H–H bonds. Lastly, the C–Cl bond in the adsorbed 4-CP is attacked by the active hydrogen atom on the surface of the catalyst, yielding the product phenol and byproduct HCl [36]. Figure 4a shows the time evolution of the yield of phenol and conversion of 4-CP over the catalyst Pd/alk-Ti_3_C_2_X_2_. It can be seen that 4-CP is completely converted to phenol within 70 min. The yield of phenol gradually increases with the increase in reaction time. With respect to product distribution, phenol is detected as the sole product in the HDC process because of the low reaction temperature and catalyst concentration [37]. As reported previously, phenol could be further hydrogenated to cyclohexanone and/or cyclohexanol with a high catalyst concentration and high reaction temperature [14,20]. With regards to the reaction kinetics, the HDC of 4-CP with Pd-based catalysts could be assumed to be a pseudo-first-order reaction considering the excess amount of H_2_ gas [36]. To this end, the reaction rate constant was determined depending on the slope of the fitting line, where ln (*C_t_*/*C*_0_*)* was plotted against reaction time (Figure 4b). The observation of a good linear correlation shows that the HDC of 4-CP catalyzed by Pd/alk-Ti_3_C_2_X_2_ is a pseudo-first-order reaction. According to the slope of the fitting line, the reaction rate constant *k* was calculated to be 5.42 × 10^−2^ min^−1^ for the catalytic HDC of 4-CP with the Pd/alk-Ti_3_C_2_X_2_ catalyst. Considering the different dosages of supported Pd catalysts, the reaction rate constant *k’*, defined as the ratio of the rate constant to the quality of the active sites, was calculated to compare the catalytic activity with the reported Pd catalyst for the HDC of 4-CP. Even though no base additives was employed, the Pd/alk-Ti_3_C_2_X_2_ catalyst exhibited a high HDC rate of 216.6 min^−1^·$g_{\mathrm{pd}}^{-1}$. This value is higher than that of the reported Pd-based catalysts, such as Pd/Al_2_O_3_ [38] and Pd/DMSNs [37], with kinetic reaction rates (k_Pd_) of 3.33 and 13.2 min^−1^·$g_{\mathrm{pd}}^{-1}$, respectively.

To kinetically study the HDC of 4-CP catalyzed by Pd/alk-Ti_3_C_2_X_2_, a set of control experiments was carried out at temperatures from 20 °C to 35 °C while keeping the other reaction conditions unchanged. As shown in Figure 4c, temperature has a positive influence on the catalytic HDC of 4-CP. The time for complete conversion of 4-CP to phenol decreases from 90 min to 35 min with the elevation of the temperature. It is noteworthy that the selectivity to phenol is still higher than 99% in all tests, indicating that the increase of reaction temperature only increases the reaction rate without changing the selectivity. The rate constant at different temperatures was calculated from the slope of the linear part of ln(*C*_t_/*C*_0_) versus time plots. Based on the slope of the Arrhenius plots in Figure 4d, the activation energy (*E*_a_) for 4-CP HDC over Pd/alk-Ti_3_C_2_X_2_ was calculated to be 51.2 kJ/mol. This value is lower than that of Pd on activated carbon (58 kJ·mol^−1^) [39], Pd/DMSNs catalyst (61.5 kJ·mol^−1^) [37], and Pd/Al pillared clays (85.8 kJ·mol^−1^) [40], corresponding to the high catalytic activity of Pd/alk-Ti_3_C_2_X_2_ for the HDC of 4-CP without any additives. The XPS results show the presence of two Pd species, Pd° and Pd^n+^, in the Pd/alk-Ti_3_C_2_X_2_ catalyst. Previous results have shown that electron-deficient and zero-valent Pd species are essential for increasing the catalytic activity of Pd-based catalysts for HDC reaction. They play different roles, activating the C–Cl bond and H_2_ in the HDC process, respectively [27,41,42,43,44]. According to the proposed dual mechanism, zero-valent Pd chemisorbed and homolytically dissociated hydrogen into the covalent adatom Pd–H [43,44]. Electrodeficient Pd^n+^ species were the centers for C–Cl bond activation via nucleophilic chloride anion abstraction [27,41]. In the present work, the presence of different Pd species including Pd° and Pd^n+^ was responsible for the high catalytic activity of Pd/alk-Ti_3_C_2_X_2_ for HDC reaction. These results confirm the findings related to the proposed dual mechanism of the HDC reaction.

### 3.3. Catalytic Hydrogenation of 4-NP

**Figure 5:**
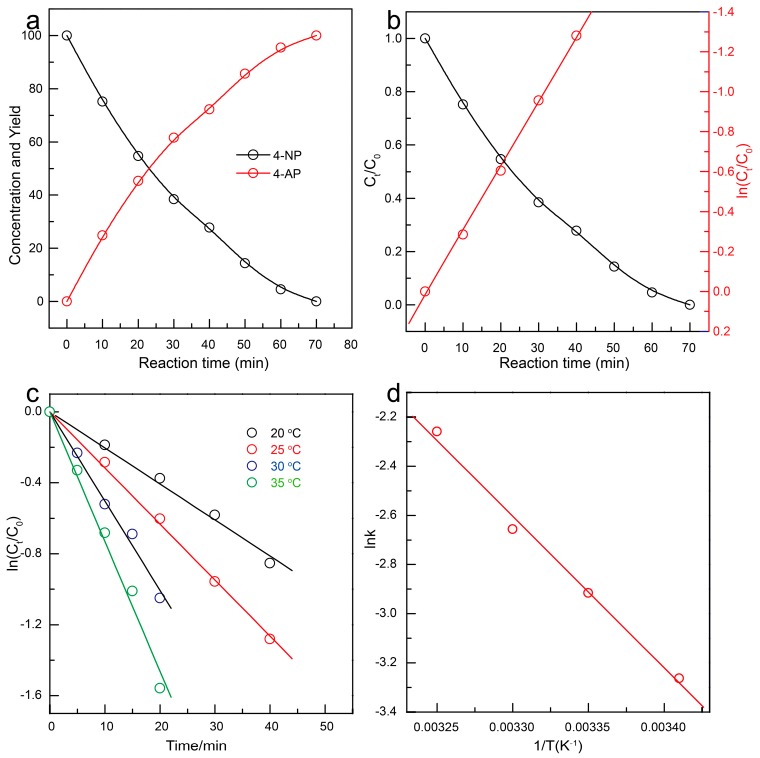
(**a**) Time evolution of the yield of 4-AP and conversion of 4-NP with the Pd/alk-Ti_3_C_2_X_2_ catalyst at 25 °C; (**b**) Plots of (*C*_t_/*C*_0_) and ln(*C*_t_/*C*_0_) vs. time for the reduction of 4-NP; (**c**) Plots of ln (*C*_t_/*C*_0_) vs. time at different temperatures; (**d**) The Arrhenius plot.

Taking into account the high catalytic activity toward the HDC of 4-CP, Pd/alk-Ti_3_C_2_X_2_ was also selected as a catalyst for the hydrogenation of 4-NP in an aqueous solution at 25 °C and balloon hydrogen pressure. Similarly, the reaction pathway for the hydrogenation of 4-NP to 4-AP can be described in Scheme 2(2). Specifically, the absorbed hydrogen is activated by the active sites of Pd dispersed on the alk-Ti_3_C_2_X_2_ surface. Two active hydrogen atoms are formed through the scission of the H–H bonds in the activated hydrogen. Finally, the –NO_2_ groups in the adsorbed 4-NP is attacked by the active hydrogen atom on the surface of the catalyst, yielding the product 4-AP. The conversion of 4-NP was monitored by GC. 4-AP was detected as the sole product; no other species were observed during the reaction process. Figure 5a shows the time-dependent conversion of 4-NP and the yield of 4-AP for the hydrogenation of 4-NP by Pd/alk-Ti_3_C_2_X_2_. Results indicate that Pd/alk-Ti_3_C_2_X_2_ also exhibits excellent catalytic activity for the hydrogenation of 4-NP to 4-AP in the presence of hydrogen gas. The complete hydrogenation of 4-NP to 4-AP was finished in 70 min. The yield of 4-NP gradually increased with the reaction time, and a 99.9% yield of 4-AP was detected. During the hydrogenation process, large amount of H_2_ was applied as compared with the concentration of 4-NP. Thus, the concentration of H_2_ can be assumed as a constant and the hydrogenation reaction is assumed to be a pseudo first-order reaction with respect to the 4-NP concentration. To this end, the reaction kinetic is expressed as ln(*C*_t_/*C*_0_) = −*kt*, in which *k* represents the apparent rate constant, and *t* is the reaction time. Figure 5b shows the plots of ln(*C*_t_/*C*_0_) versus reaction time *t*. The fitting straight line indicates that the hydrogenation of 4-NP is a pseudo-first-order reaction by using hydrogen gas as the reducing agent. According to the slope of the fitting line, the reaction rate constant *k* for the catalytic reduction of 4-NP by Pd/alk-Ti_3_C_2_X_2_ is 3.16 × 10^−2^ min^−1^, corresponding to a high initial reaction rate of 126.3 min^−1^·$g_{\mathrm{pd}}^{-1}$. Figure 5c shows the hydrogenation of 4-NP over Pd/alk-Ti_3_C_2_X_2_ catalyst at different temperatures. An increase of 3.6-fold times in the reaction rate constant was observed with the elevation of temperature from 20 °C to 35 °C. The rate constant *k* at different temperatures was evaluated from the slope of the linear part of each plot from Figure 5c. The Arrhenius plots of ln*k* versus 1/T for the catalyst are shown in Figure 5d. The activation energy was calculated to be 67.5 kJ/mol.

**Figure 6:**
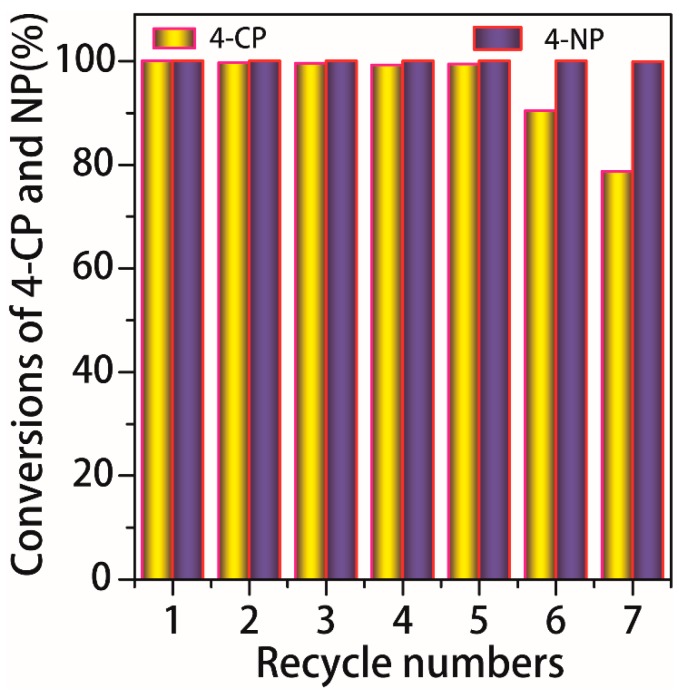
Recyclability of Pd/alk-Ti_3_C_2_X_2_ for the HDC of 4-CP and the hydrogenation of 4-NP at 25 °C.

Stability and recyclability of heterogeneous catalyst are of great significance in terms of practical applications. Therefore, the stability and recyclability of catalyst Pd/alk-Ti_3_C_2_X_2_ for the HDC of 4-CP and the hydrogenation of 4-NP were studied, respectively. After the completion of the first run, the solid catalyst was recovered from the mixture by centrifugation, and the supernatant liquid was collected for detecting the conversion efficiency by GC. The recovered black solid was treated with successive washing cycles with water and ethanol and transferred into the reactor for the next run with the introduction of fresh 4-CP and 4-NP. Results shown in Figure 6 indicate that the Pd/alk-Ti_3_C_2_X_2_ catalyst is quite stable and can be recycled seven times without significant loss of removal efficiency for the hydrogenation of NP. With regard to the recyclability of Pd/alk-Ti_3_C_2_X_2_ for the HDC of 4-CP, the catalyst can be recycled five times without the loss of any catalytic activity. These results show that Pd/alk-Ti_3_C_2_X_2_ has high durability toward deactivation during the recycling process, which has never been reported before.

## 4. Conclusions

In summary, we have developed a simple method to prepare a Pd/alk-Ti_3_C_2_X_2_ catalyst with large surface area and abundant surface terminations through the alkalization of the support. These merits endow the as-prepared Pd/alk-Ti_3_C_2_X_2_ with superior catalytic activities toward the HDC of 4-CP and the hydrogenation of 4-NP under mild conditions. The Pd/alk-Ti_3_C_2_X_2_ catalyst showed very high initial reaction rates of 216.6 and 126.3 min^−1^·$g_{\mathrm{pd}}^{-1}$ for the HDC of 4-CP without alkali additive and hydrogenation of 4-NP without the reducing agent, respectively. Moreover, the Pd/alk-Ti_3_C_2_X_2_ catalyst was quite stable and could be recycled five or seven times without significant loss of catalytic efficiency in the HDC of 4-CP and the hydrogenation of 4-NP, respectively. The high catalytic activity and reusability of Pd/alk-Ti_3_C_2_X_2_ make it a promising candidate for various catalytic applications.
